# An improved grey wolf optimization-based MPPT algorithm for photovoltaic systems under partial shading conditions

**DOI:** 10.1038/s41598-026-45860-z

**Published:** 2026-04-09

**Authors:** Resat Celikel, Omur Aydogmus, Musa Yilmaz

**Affiliations:** 1 Department of Mechatronics Engineering, Firat University, 23200 Elazig, Turkey; 2 Department of Electrical and Electronics Engineering, Batman University, 72100 Batman, Turkey; 3 Center for Environmental Research and Technology, Bourns College of Engineering, University of California at Riverside, Riverside, CA 92521 USA

**Keywords:** PV system, Grey wolf optimization, MPPT, Partial shading

## Abstract

Maximum Power Point Tracking (MPPT) algorithms, which are employed to extract the maximum power from photovoltaic (PV) systems, exhibit different performance characteristics under uniform irradiance and partial shading conditions (PSC) arising from nonuniform solar irradiance distribution on PV panels. Under PSC, the performance of conventional MPPT algorithms becomes inadequate, leading to increased interest in optimization-based approaches. In this study, the Grey Wolf Optimization (GWO) algorithm, commonly used in MPPT applications, was modified, and an Improved Grey Wolf Optimization (IGWO) algorithm was proposed. A PV system model consisting of four series-connected PV panels and a boost converter was developed in the MATLAB/Simulink environment to evaluate the performance of the proposed algorithm. The effectiveness of the algorithm was tested under nine distinct and complex PSC scenarios. The results obtained under these nine PSC cases were analyzed through comparisons of the proposed IGWO algorithm with GWO, the Cuckoo Search Algorithm (CSA), and the Flower Pollination Algorithm (FPA). The results demonstrate that the IGWO algorithm achieves the highest mean maximum power and exhibits superior MPPT performance compared to the other algorithms, with a mean tracking efficiency of 98.34%.

## Introduction

Global warming, environmental pollution, the depletion of carbon-based fuel reserves, and the steadily increasing energy demand have significantly increased interest in renewable energy sources. Among renewable energy sources, solar energy stands out due to its sustainability and wide applicability. Photovoltaic (PV) panels generate different power levels depending on variable atmospheric conditions such as irradiance and temperature. The relatively low efficiency of PV panels and the requirement to operate at maximum power under varying operating conditions necessitate the use of effective control and tracking methods. Accordingly, Maximum Power Point Tracking (MPPT) algorithms have been developed to maximize the power extracted from PV systems^1,2^.

In PV systems, PV panels are connected in series and parallel configurations to achieve the desired power and voltage levels. When all panels in a PV system are subjected to identical temperature and irradiance conditions, the system is considered to operate under uniform irradiance, whereas exposure of panels to different irradiance levels is referred to as partial shading conditions (PSC). Under uniform irradiance conditions, conventional MPPT algorithms such as Perturb and Observe (P&O), Incremental Conductance (InC), and the 0.8 V_oc_ method provide effective and reliable performance^3^. However, MPPT algorithms are expected to rapidly reach the maximum power point during transient conditions while minimizing power oscillations under steady-state operation. To satisfy these requirements, advanced MPPT approaches have been proposed in the literature, including Fuzzy Logic^4,5^, Artificial Neural Networks (ANNs)^6,7^, modified P&O^8,9^, and modified InC^10,11^.

Achieving maximum power in PV systems operating under PSC constitutes a significant challenge. The primary reason for this difficulty is that, under PSC, multiple maximum power points can occur at different voltage levels depending on the number of PV panels. Among these points, only one corresponds to the highest power, known as the Global Maximum Power Point (GMPP), and operating the PV system at this point is critical for maximizing power generation. The remaining maximum power points are defined as Local Maximum Power Points (LMPPs), and operation at these points results in undesirable power losses. To achieve maximum power under PSC, modified conventional MPPT algorithms have been proposed in the literature^12,13^. In addition, scanning-based MPPT algorithms, similar in principle to conventional MPPT approaches, have also been reported in recent studies. MPPT algorithms based on voltage scanning and voltage transient behavior stand out due to their high tracking speed and efficiency^14,15^. While scanning-based algorithms offer the advantage of being independent of PV system parameters, high-speed and high-efficiency methods such as voltage segmentation and the modified 0.8 V_oc_ are known to operate in a parameter-dependent manner with respect to PV panel characteristics^16,17^.

Interest in optimization-based MPPT algorithms has increased in recent years, primarily because these algorithms offer a significant advantage over conventional methods by largely eliminating power oscillations under steady-state conditions. Moreover, these approaches are capable of achieving high performance under both uniform irradiance and partial shading conditions (PSC), thereby reducing the need for separate PSC detection mechanisms. It has been demonstrated in the literature that Particle Swarm Optimization (PSO)-based MPPT algorithms provide superior performance under both uniform and PSC conditions compared to conventional hill-climbing methods, while significantly reducing steady-state power fluctuations^18^. The Cuckoo Search Algorithm (CSA) is another optimization algorithm widely used in MPPT applications, its effectiveness has been validated in numerous studies. CSA can be applied independently or in combination with the Golden Section Search (GSS) algorithm to achieve faster maximum power point tracking^19,20^. Unlike swarm-based algorithms, the Flower Pollination Algorithm (FPA) is inspired by the natural pollination mechanism of flowers and has been extensively employed in the literature to extract maximum power from PV systems^21,22^.

In recent years, a wide range of optimization-based approaches has been proposed for MPPT applications, including the honey badger algorithm^23^, seagull optimization algorithm^24^, coot optimization algorithm^25^, dung beetle optimization algorithm^26^, zebra optimization algorithm^27^, search and rescue optimization algorithm^28^, horse herd optimization algorithm^29^, salp swarm optimization algorithm^30^, roach infestation optimization algorithm^31^, falcon optimization algorithm^32^, arithmetic optimization algorithm^33^, and musical chairs algorithm^34^. The primary objective of these algorithms is to reach the maximum power point with high efficiency and short tracking time under complex PSC scenarios. However, in practical applications, low computational burden is of critical importance for microcontroller-based systems. Reducing the computational complexity of MPPT algorithms enables cost reduction through the use of simpler hardware.

The Grey Wolf Optimization (GWO) algorithm is one of the early optimization-based methods developed for MPPT applications and has become an important benchmark approach frequently used to compare the performance of newly developed algorithms in the literature. GWO models the leadership hierarchy and hunting behavior of grey wolves in nature^35^. Although the GWO algorithm has demonstrated successful results in identifying the maximum power point in PV systems, further studies have been required to improve its tracking speed. In this context, a modified GWO algorithm in which the convergence factor a was adjusted achieved an approximately 45% improvement in tracking time^36^. Similarly, enhanced GWO algorithms, in which the convergence factor a was restructured using trigonometric expressions, were comparatively evaluated in combination with the Salp Swarm Optimization algorithm^37,38^. The results indicate that the improved GWO algorithms provide significant advantages, particularly for PV systems operating under PSC.

In another study, a newly designed two-stage MPPT algorithm combined the GWO and P&O algorithms, resulting in a substantial improvement in tracking time^39^. Furthermore, MPPT approaches that hybridize GWO and the Whale Optimization Algorithm (WOA) with the P&O method were proposed, and their performance was compared through simulations conducted under uniform irradiance conditions and using real field data^40^. A high-efficiency hybrid MPPT algorithm, developed by integrating GWO with the Equilibrium Optimizer algorithm, was experimentally compared with Particle Swarm Optimization (PSO), WOA, and FPA methods^41^. Similarly, high-efficiency hybrid MPPT algorithms based on CSA-GWO and PSO-GWO combinations were developed, and their effectiveness was validated under different PSC scenarios^42,43^. Finally, in a recently developed GWO-based MPPT algorithm, tracking efficiency was further improved, and the experimentally obtained results were presented graphically^44^.

In this study, a novel MPPT algorithm based on Improved Grey Wolf Optimization (IGWO) is proposed by redefining the operating boundaries of the conventional GWO algorithm. The proposed IGWO algorithm is designed to operate over a total of 20 iterations. In the GWO algorithm, if the targeted maximum power is reached within the first 10 iterations, the process is terminated; otherwise, when the desired power variation is not achieved, the duty cycle corresponding to the highest power obtained at the end of the 10th iteration is used as the initial reference, and the algorithm is restarted. With this approach, the search space of the GWO algorithm is constrained, aiming to reach the maximum power point more rapidly within a narrower operating range.

## PV system modelling

**Fig. 1:**
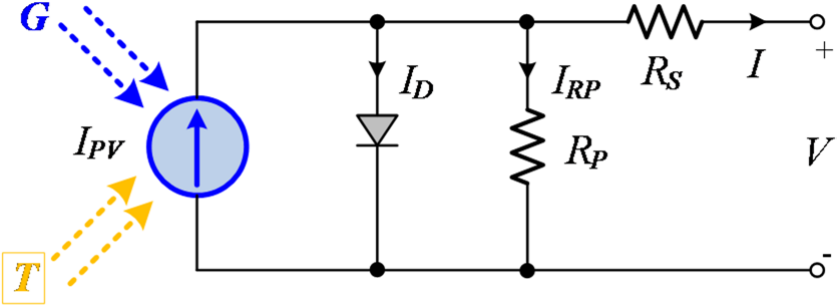
Single PV cell modelling.

A PV cell can be represented by an equivalent circuit model consisting of a diode, two resistors, and a current source, as shown in Fig. 1^45,46^. By connecting PV cells in series and parallel, PV panels are formed, and multiple panels combined together constitute PV arrays. In this study, a PV system model is developed in the MATLAB/Simulink environment by connecting four SunPower SPR-X19-240 PV panels in series.

Each PV panel produces approximately 240 W of power at the maximum power point under nominal test conditions (1000 W/m^2^, 25 °C), resulting in a total nominal power of 960 W for the four-panel system. A boost converter is connected to the output of the PV system to supply the load and enable the implementation of MPPT algorithms. To operate the optimization-based MPPT algorithms, the current and voltage values of the PV system are measured. The parameters of the PV panels and the boost converter are presented in Table 1.

**Table 1:** PV system parameters.

SunPower SPR-X19-240 parameters			Boost converter parameters		
$P_{mp}$	240.165	W	*C*1	50	µF
$V_{OC}$	48.6	V	*C*2	100	µF
$I_{SC}$	6.3	A	*L*	100	µH
$V_{mp}$	40.5	V	$f_{sw}$	40	kHz
$I_{mp}$	5.93	A	$f_{sample}$	1	µs
$R_{sh}$	325.92	*Ω*	*R*	220	Ω
$R_{s}$	0.47672	*Ω*			

**Fig. 2:**
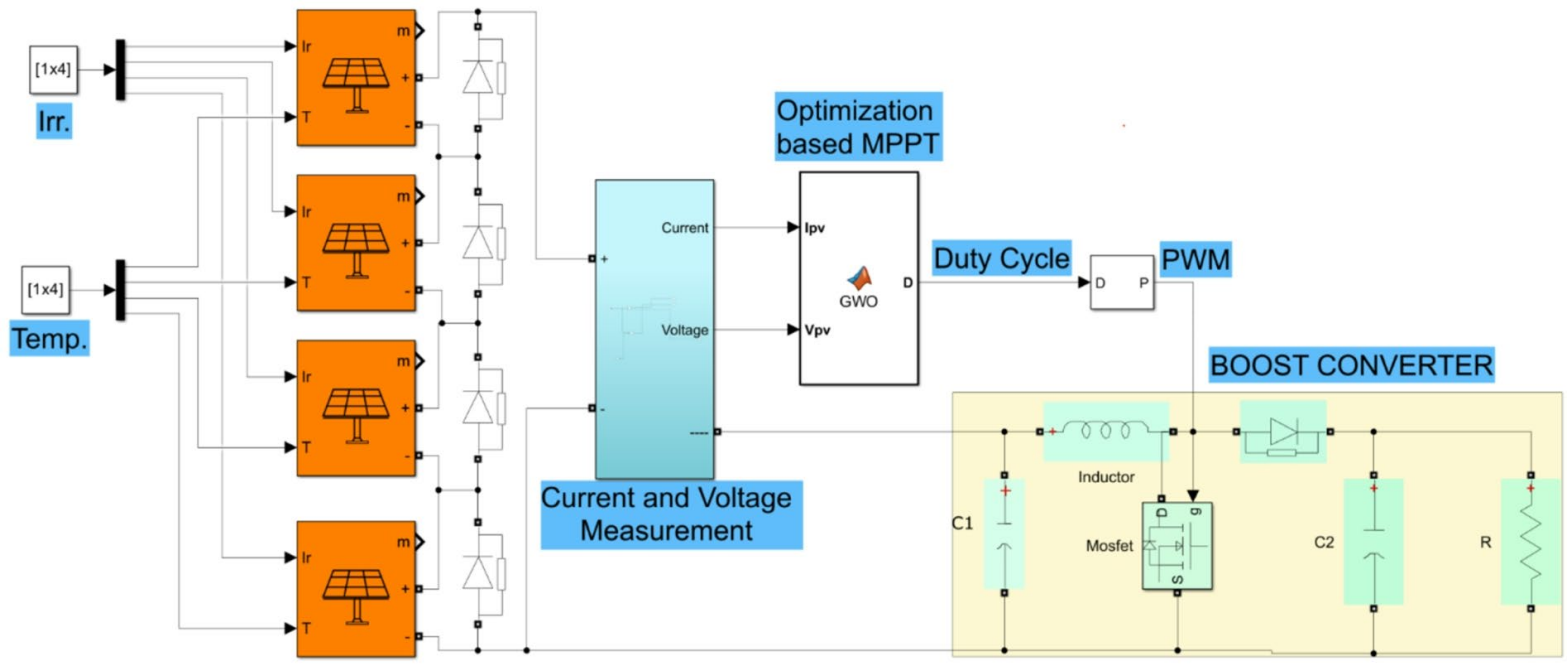
MATLAB blocks of the PV system.

In the PV system simulation, the sampling time is set to 1 µs, while the switching frequency of the boost converter is 40 kHz. The MATLAB/Simulink blocks of the PV system simulation are shown in Fig. 2.

**Fig. 3:**
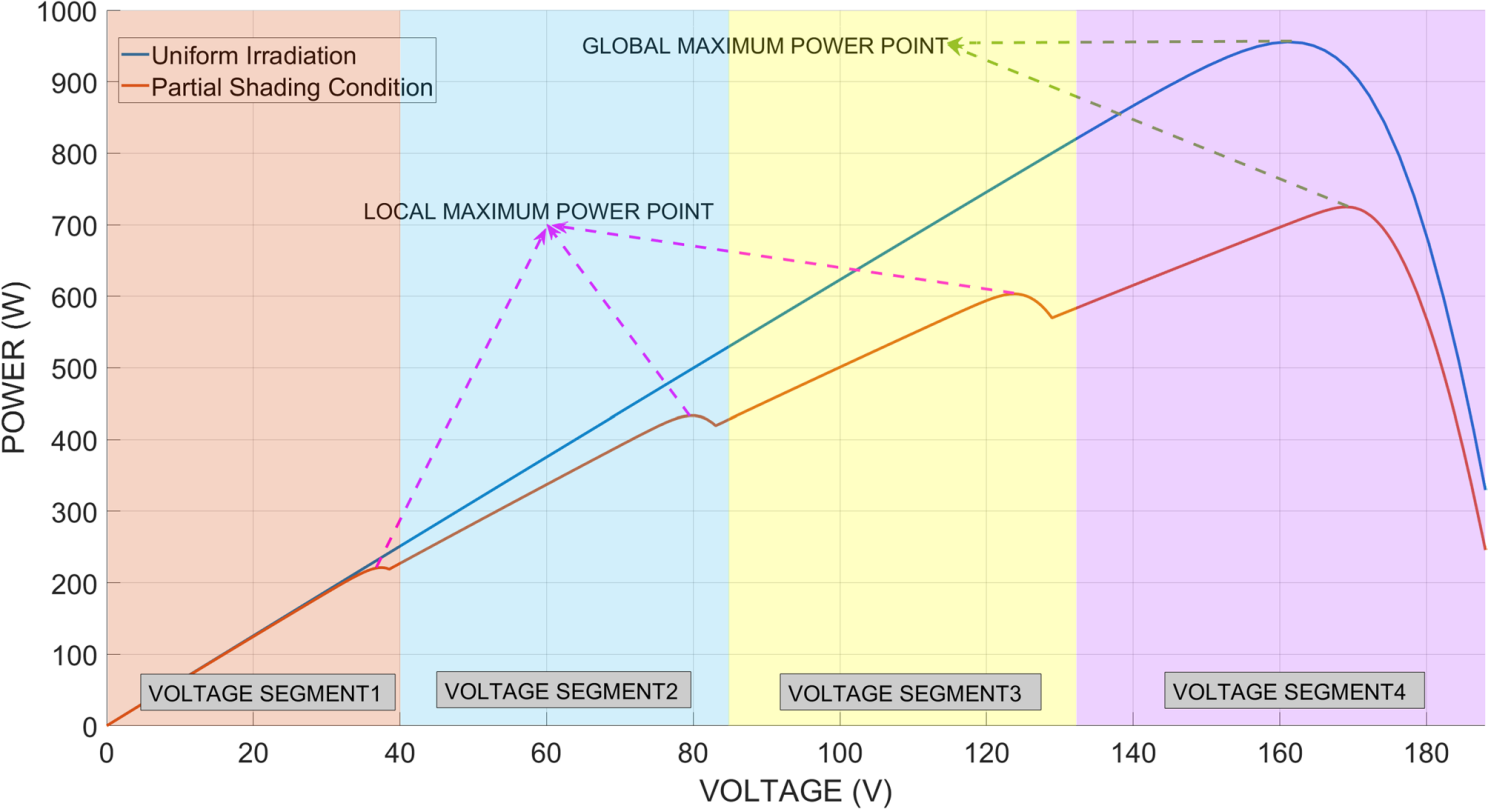
P–V curves of the PV system under uniform irradiance and PSC.

When all PV panels in a PV system are subjected to the same irradiance level, the operating condition is referred to as uniform irradiance. The effectiveness of conventional and modified MPPT algorithms under uniform irradiance conditions has been well established. When PV panels are exposed to irradiance levels of varying magnitudes, partial shading conditions (PSC) occur^47^. The P–V curves obtained under uniform irradiance and PSC conditions are shown in Fig. 3.

As shown in Fig. 3, multiple maximum power points occur under PSC. Among these points, only one corresponds to the GMPP, while the others are LMPPs. Conventional MPPT algorithms may converge to LMPPs, which leads to unsuccessful operation under PSC. Consequently, the need to develop new and advanced MPPT algorithms has emerged. Another important aspect to consider is that the number of potential maximum power points formed in different voltage regions increases with the number of series-connected PV panels.

## IGWO-based MPPT algorithm

The GWO algorithm, proposed by Mirjalili et al.^48^, mathematically models the hierarchical social structure and hunting strategies of grey wolves. In nature, grey wolves are apex predators that live and hunt in packs. To emulate this leadership hierarchy, four types of wolves are defined in the GWO algorithm: alpha (α), beta (β), delta (δ), and omega (ω). In a grey wolf pack, the alpha leads the group by directing activities such as hunting and migration. If the alpha becomes ineffective, leadership is assumed by the beta. The delta supports both the alpha and beta, while the omega represents the remaining members of the pack. The GWO algorithm is structured according to this hierarchical order. Accordingly, the alpha (α) symbolizes the optimal solution, reflecting the wolves’ leadership hierarchy. The beta (β) and delta (δ) represent the second and third best solutions, respectively, while the omega (ω) denotes all other candidate solutions. During hunting, grey wolves exhibit encircling behavior around their prey. The iterative process begins at the onset of hunting; therefore, the α, β, and δ wolves guide the remaining wolves (search agents) to surround the prey. This encircling behavior is mathematically expressed in Eq. (1).

1 $$\vec{X}\left( {t + 1} \right) = \left| {\vec{X}_{p} \left( t \right) - \overrightarrow {A.} \vec{D}} \right|$$

Here $\vec{X}$ represents the position of the search agents, $\vec{X}_{p}$ denotes the prey position, and $\vec{A}$ is the coefficient vector at the $\left( {t + 1} \right){\mathrm{th}}$ iteration. The coefficient $\vec{D}$ is defined in Eq. (2).

2 $$\vec{D} = \left| {\vec{C}.\vec{X}_{p} \left( t \right) - \vec{X}_{p} \left( t \right)} \right|$$

Here, the parameter vectors $\vec{A}$ and $\vec{C}$ are obtained using the randomly generated vectors $\vec{r}_{1}$ and $\overrightarrow { r}_{2}$ whose elements are randomly selected within the interval [0, 1], as shown in Eqs. (3) and (4).

3 $$\vec{A} = 2\vec{a}.\vec{r}_{1} - \vec{a}$$

4 $$\vec{C} = 2\overrightarrow {.r}_{2}$$

Here, the components of $\vec{a}$ decrease linearly from 2 to 0 over the iterations.

During the hunting process, grey wolves update their positions by considering the location of the prey. The α, β, and δ wolves guide the ω wolves toward potential prey locations, thereby coordinating their movements to maximize hunting efficiency. The following equations describe the position-update mechanism through which the pack collectively tracks the prey and maximizes hunting success.

5 $$\begin{array}{*{20}c} {\overrightarrow {{D_{\alpha } }} = \left| {\overrightarrow {{C_{1}^{t} }} .\overrightarrow {{X_{\alpha }^{t} }} - X^{t} } \right|} \\ {\overrightarrow {{D_{\beta } }} = \left| {\overrightarrow {{C_{1}^{t} }} .\overrightarrow {{X_{\beta }^{t} }} - X^{t} } \right|} \\ {\overrightarrow {{D_{\delta } }} = \left| {\overrightarrow {{C_{1}^{t} }} .\overrightarrow {{X_{\delta }^{t} }} - X^{t} } \right|} \\ \end{array}$$

6 $$\begin{array}{*{20}c} {\overrightarrow {{X_{1} }} = \left| {\overrightarrow {{X_{\alpha }^{t} }} - A_{1}^{t} .\overrightarrow {{D_{\alpha }^{t} }} } \right|} \\ {\overrightarrow {{X_{2} }} = \left| {\overrightarrow {{X_{\beta }^{t} }} - A_{2}^{t} .\overrightarrow {{D_{\beta }^{t} }} } \right|} \\ {\overrightarrow {{X_{3} }} = \left| {\overrightarrow {{X_{\delta }^{t} }} - A_{2}^{t} .\overrightarrow {{D_{\delta }^{t} }} } \right|} \\ \end{array}$$

7 $$X^{t + 1} = \frac{{X_{1}^{t} + X_{2}^{t} + X_{3}^{t} }}{3}$$

The position vector is modified by $\vec{a}$ at each iteration to guide the omega-type wolves either toward or away from the prey. The vector $\vec{a}$ decreases from 2 to 0 after each iteration, as expressed in Eq. (8).

8 $$\vec{a} = 2\left( {1 - \frac{t}{N}} \right)$$

Here, *t* represents the current iteration number, while *N* denotes the total number of iterations.

In the IGWO method, upon reaching a certain iteration, the initial conditions or boundaries of the search space are redefined, and the GWO algorithm is restarted to improve hunting performance. Initially, the GWO algorithm is run for 10 iterations. If, at the end of the 10th iteration, the change in power is very small ($\Delta P_{pv} < 5$), it is considered that the maximum power has been approached, and the algorithm is terminated. Otherwise, the position corresponding to the closest approach to the prey is determined, and the GWO algorithm is restarted. In the second step, the position obtained in the first step ($D_{new}$) is used to define the initial conditions, as shown in Eq. (9). By narrowing the search area in this second step, the success rate of locating the maximum power point is increased.

9 $$\begin{array}{*{20}c} {D_{2\_1} = D_{new} + \left[ {\left( {D_{4} + D_{1} } \right) \times 0.2} \right]} \\ {\begin{array}{*{20}c} {D_{2\_2} = D_{new} - \left[ {\left( {D_{4} + D_{1} } \right) \times 0.2} \right]} \\ {D_{2\_3} = D_{new} + \left[ {\left( {D_{4} + D_{2} } \right) \times 0.2} \right]} \\ {D_{2\_4} = D_{new} - D_{1} } \\ \end{array} } \\ \end{array}$$

**Fig. 4:**
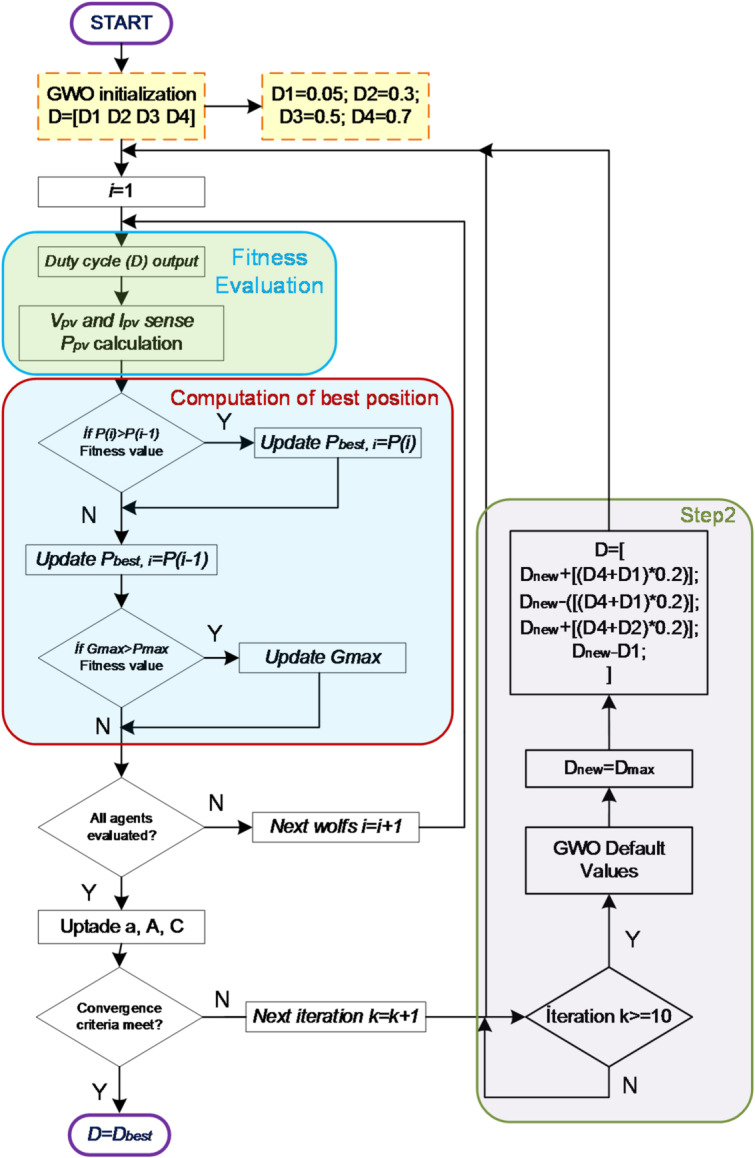
Flowchart of IGWO algorithm.

Here, $D_{1} , D_{2} , D_{3} ,$ and $D_{4}$ represent the initital duty cycles determined during the first execution of the GWO algorithm and are set to 0.05, 0.3, 0.5, and 0.7, respectively. The flowchart of the IGWO algorithm is shown in Fig. 4.

In GWO algorithm, if convergence toward the maximum power cannot be achieved within a specified number of iterations under steady-state conditions according to the expression given in Eq. (10) the algorithm is terminated, and the best position attained up to that point is accepted as the optimization result. In this study, after the 10th iteration, the search space was narrowed to achieve faster convergence and higher efficiency. The 10th iteration threshold was determined through extensive trial-and-error analyses. Restarting the algorithm before the 10th iteration led to unfavorable results in certain scenarios. This is because, during the initial startup phase, the assigned duty cycles operate temporarily under transient conditions, which may mislead the algorithm. To mitigate the influence of transient states during initial operation and under dynamic atmospheric conditions, the number of iterations was selected such that each duty cycle (D1–D2–D3–D4) was applied at least twice.

10 $$\left| {P_{pv\_new} - P_{pv\_last } } \right| \le \Delta_{pv} , \;\Delta_{pv} = 5$$

After the 10th iteration, redefining the initial duty cycles using a simple computational technique such that they oscillate around the newly assigned starting value facilitates convergence toward the maximum power point and enhances the algorithm’s efficiency without increasing the number of iterations. In the proposed method, the values added to and subtracted from the new initial value are obtained using 20% of the sum of the first assigned maximum and minimum duty cycles, 20% of the sum of the second and last duty cycles, and the first duty cycle. In the second stage, the newly generated initial duty cycles were associated with the initial state, and the reuse of identical values was prevented. Consequently, after the 10th iteration, the power values oscillate around the newly assigned initial point, and the subsequent position is determined based on the power levels obtained from the preceding and succeeding duty cycles.

If, after the second stage and up to the 10th iteration, the convergence criterion toward the maximum power specified in Eq. (10) is not satisfied, the algorithm is terminated, and the highest power value obtained in the second stage is accepted as the optimization result. Even in cases where convergence to the maximum power is not fully achieved, the IGWO algorithm continues the search process around the maximum power region, as it utilizes the duty cycle transferred from the first stage in the second stage. Consequently, the probability of accurately identifying the maximum power point is increased.

## Simulation results

**Fig. 5:**
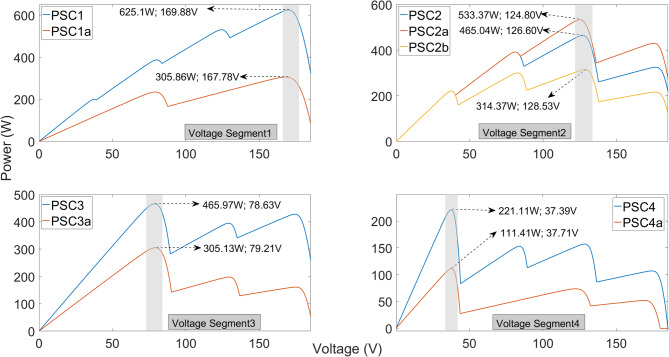
Nine different PSCs used to test proposed algorithm.

MPPT algorithms are expected to reach the maximum power rapidly and operate at high efficiency under PSC in PV systems. Therefore, the proposed algorithm is tested under nine different PSC scenarios, as illustrated in Fig. 5. The irradiation levels applied to each PV panel for generating the P–V curves illustrated in Fig. 5 are presented in Table 2. All partial shading condition (PSC) scenarios were established at a constant temperature of 25 °C.

**Table 2:** Irradiation levels in PV panels in PSC scenarios.

	Irradiation Levels in Partial Shading Conditions (W/m^2^)								
	PSC1	PSC1a	PSC2	PSC2a	PSC2b	PSC3	PSC3a	PSC4	PSC4a
PV1	600	500	600	700	200	1000	650	200	500
PV2	700	500	800	800	1000	1000	650	1000	100
PV3	800	300	1000	400	400	400	250	100	100
PV4	900	300	300	1000	600	500	150	300	50

A detailed examination of Fig. 5 reveals PSCs in which maximum power points of different magnitudes occur across four distinct voltage regions. Moreover, as observed in PSC2b and PSC3, there are challenging scenarios in which multiple power points within the same PSC scenario produce very similar power levels in different voltage regions. Similarly, the maximum power points in PSC1a and PSC3a occur at different voltage regions but have approximately the same power values. In PSC2, PSC2a, and PSC2b, two of the maximum power points are close to each other, while the third is lower, with all occurring within the same voltage region. The PSCs include P–V curves with two, three, or four distinct maximum power values. To evaluate the performance of the proposed algorithm, challenging PSC scenarios were created, and the proposed IGWO method was compared with the GWO, FPA, and CSA algorithms. All methods were tested under identical conditions, with the GWO and IGWO algorithms completing the same number of iterations. Each iteration was applied at equal time intervals. Simulation time and sampling time were taken as equal for all methods. Optimization stopping criteria were limited by Eq. 10 and the maximum number of iterations for GWO and IGWO, while they were limited by Eq. 10 for FPA and CSA. Important parameters of the optimization algorithms are given in Table 3.

**Table 3:** Parameters of optimization algorithms.

Optimization method	Parameters	Value
GWO	Max. iteration	20
	Search agents	4
	$\vec{a}$	Linearly decreased from 2 to 0
	Stopping criteria	Maximum iteration
		$$\left| {P_{pv\_new} - P_{pv\_last } } \right| \le \Delta_{pv}$$
FPA	Pollen Number	4
	Probability (P)	0.8
	Levy exponent ($\lambda$)	1.5
	Stopping criteria	$\left| {P_{pv\_new} - P_{pv\_last } } \right| \le \Delta_{pv}$
CSA	Nests numners	4
	Discovery rate (*p*_*a*_)	0.25
	Levy exponent ($\lambda$)	1.5
	Stopping criteria	$\left| {P_{pv\_new} - P_{pv\_last } } \right| \le \Delta_{pv}$
IGWO	Max. iteration	20
	Search agents	4
	$\vec{a}$	Linearly decreased from 2 to 0
	Stopping criteria	Maximum iteration
		$$\left| {P_{pv\_new} - P_{pv\_last } } \right| \le \Delta_{pv}$$

**Fig. 6:**
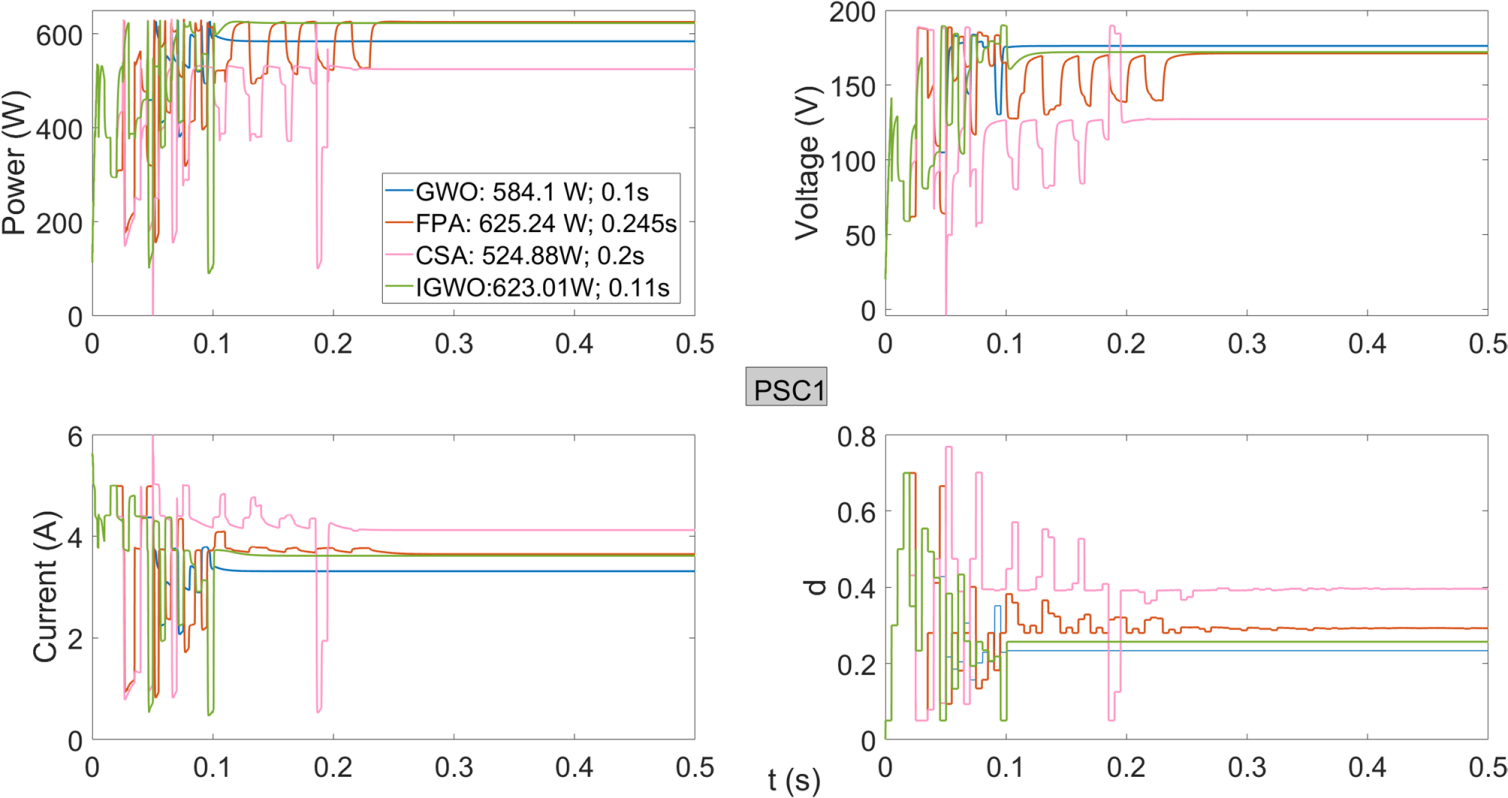
Power, current, voltage, and duty cycles obtained using GWO, FPA, CSA, and IGWO algorithms for PV system under PSC1 conditions.

As shown in Fig. 6, under PSC1, the PV system achieves a maximum power of 625.24 W using the FPA method. The IGWO algorithm produces a power of 623.01 W, which is very close to the FPA result. Although the GWO algorithm is faster, it achieves only 584.1 W, demonstrating lower performance. While the FPA algorithm generates slightly higher power than IGWO, its tracking speed of 0.245 s is considerably slower.

**Fig. 7:**
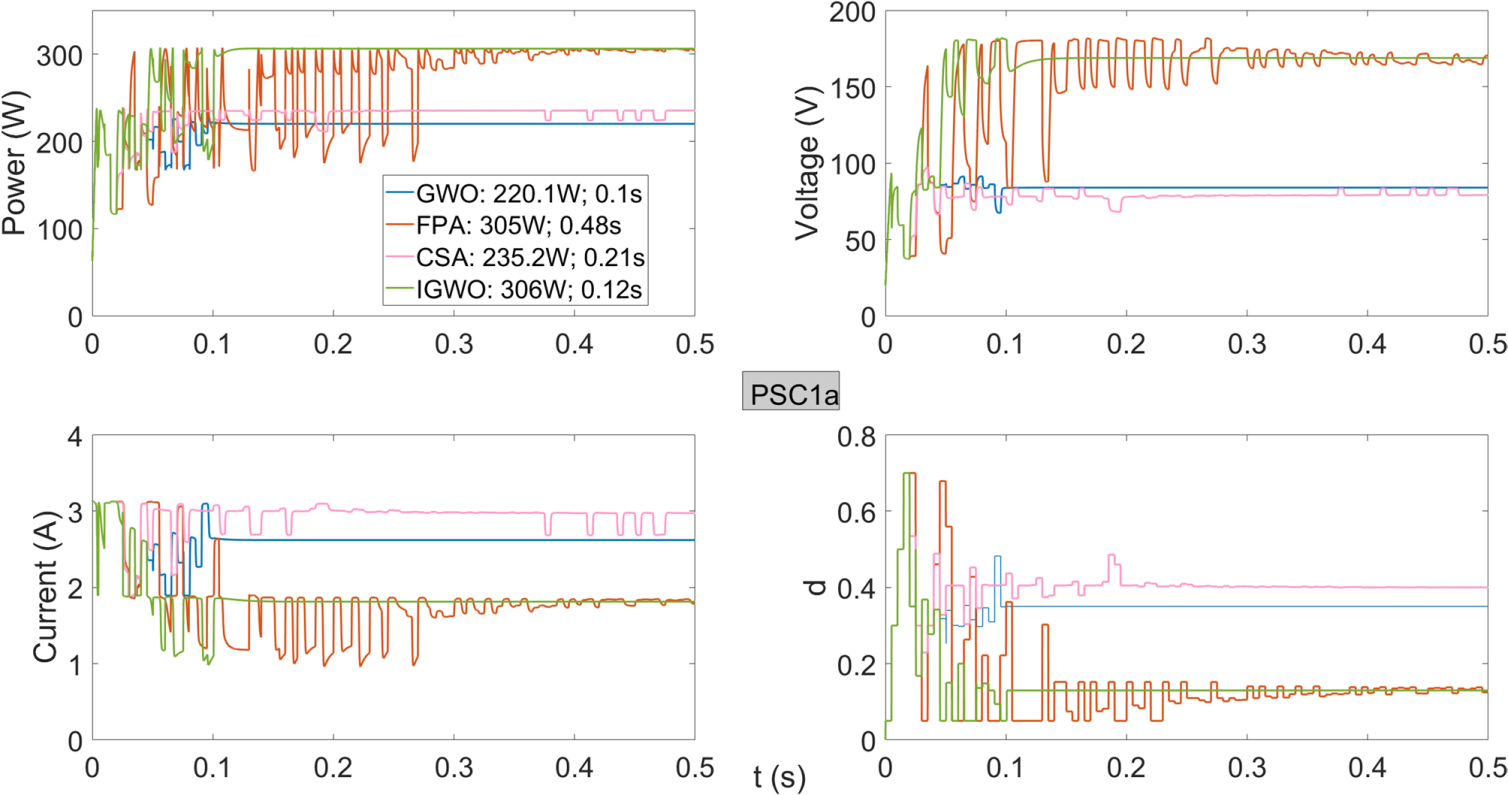
Power, current, voltage, and duty cycles obtained using GWO, FPA, CSA, and IGWO algorithms for PV system under PSC1a conditions.

As observed in Fig. 7, the IGWO algorithm achieves the highest power of 306 W while reaching the maximum power in approximately the same time as the GWO algorithm. Although the GWO algorithm has a high tracking speed, it delivers the lowest output power of 220.1 W from the system. The FPA algorithm closely approaches the maximum power but demonstrates a very low tracking speed of approximately 0.5 s. The CSA algorithm shows poor performance in both tracking speed and maximum power output. When examining the maximum power obtained under PSC1 and PSC1a conditions, both IGWO and FPA achieve the highest efficiency at the GMPP voltage region; however, the tracking time of FPA is significantly longer than that of IGWO.

**Fig. 8:**
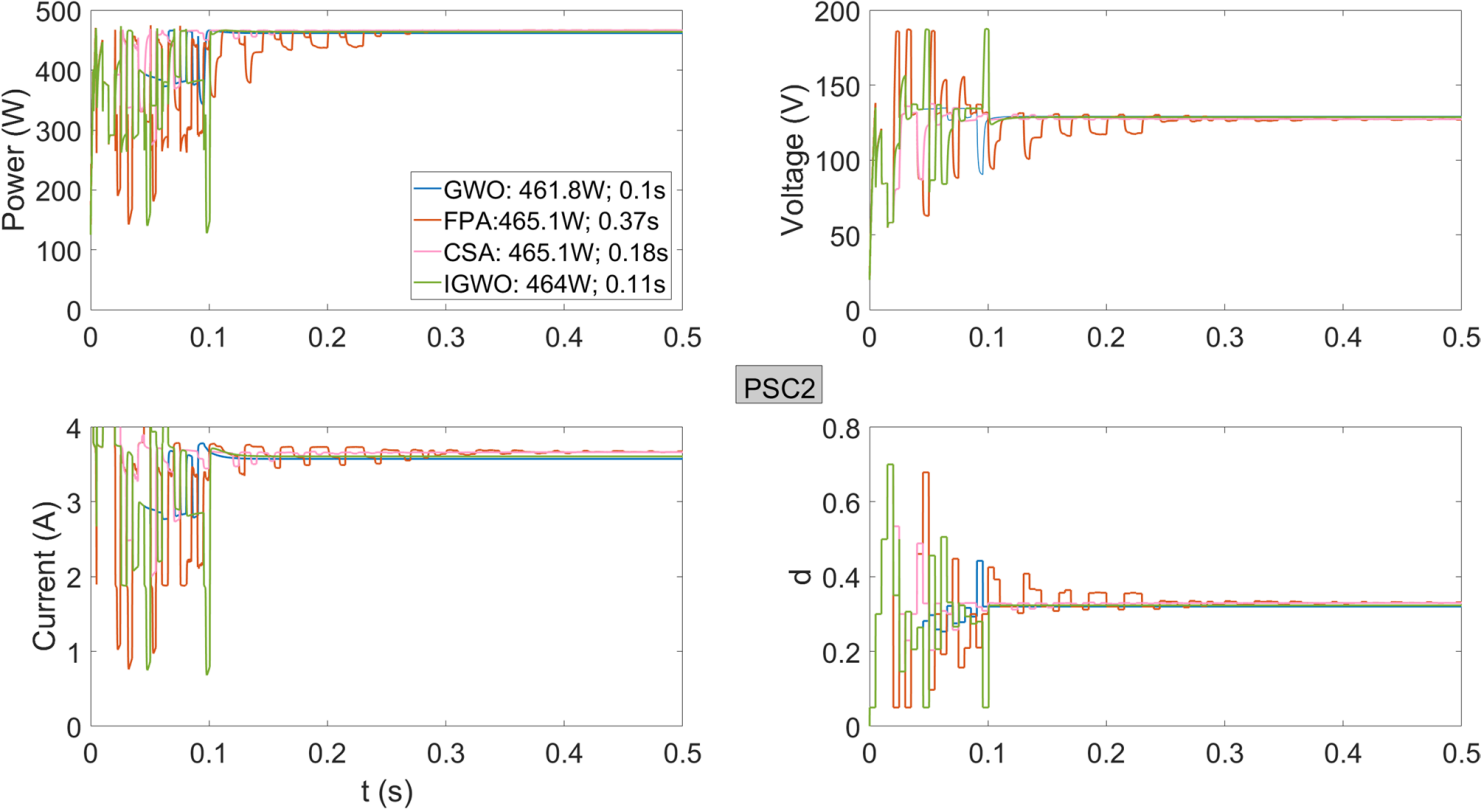
Power, current, voltage, and duty cycles obtained using GWO, FPA, CSA, and IGWO algorithms for PV system under PSC2 conditions.

In Fig. 8, the algorithms were executed for the GMPP occurring in another voltage region. Except for the GWO algorithm, all other methods produced approximately the same power. Specifically, FPA, CSA, and IGWO achieved powers of 465.1 W, 465.1 W, and 464 W, respectively, while GWO delivered 461.8 W. The IGWO algorithm reached the maximum power faster than CSA and FPA, with a tracking speed of 0.11 s.

**Fig. 9:**
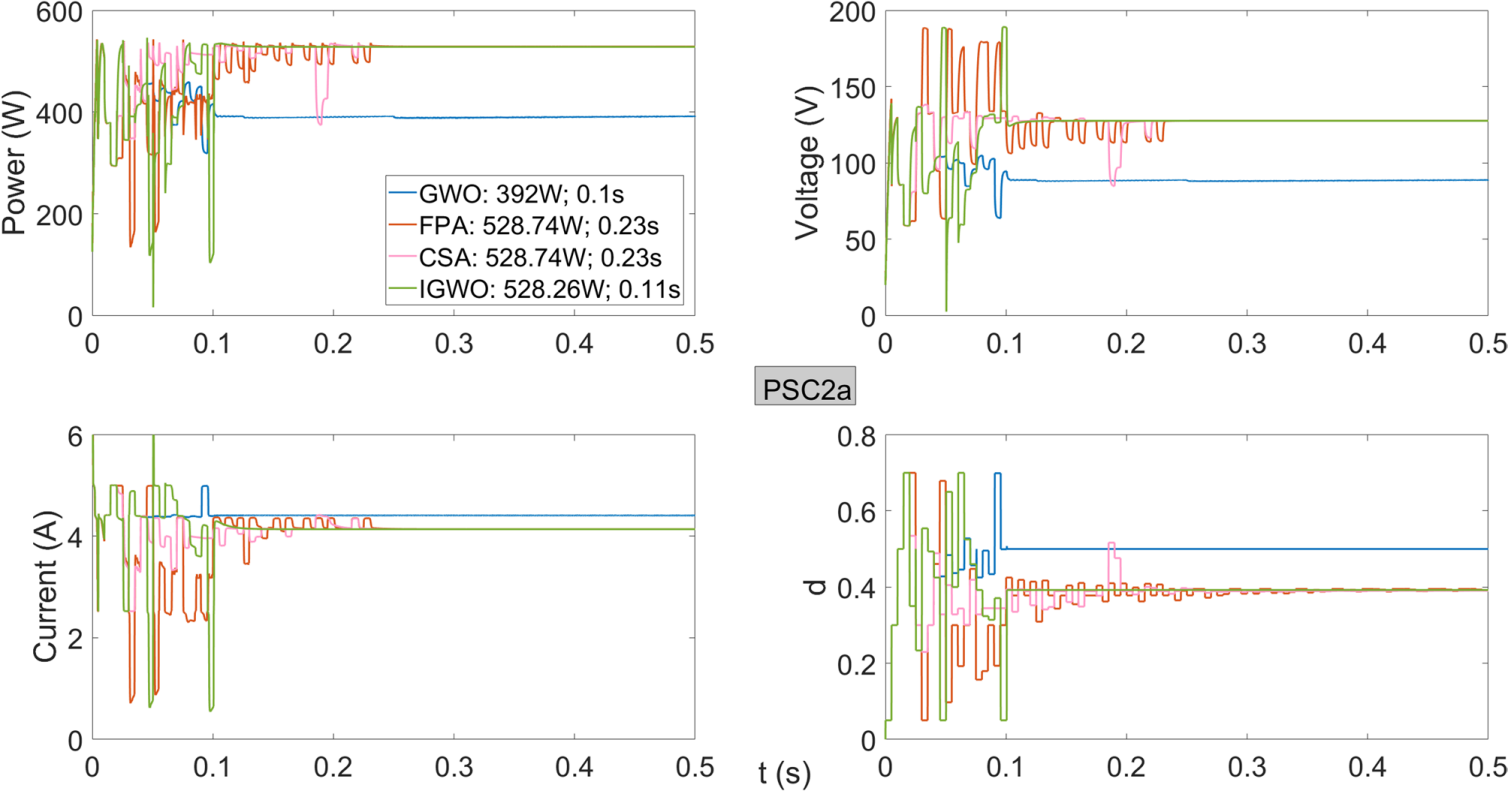
Power, current, voltage, and duty cycles obtained using GWO, FPA, CSA, and IGWO algorithms for PV system under PSC2a conditions.

As shown in Fig. 9, the performance of the algorithms under PSC2 is similar, except for GWO. The GWO algorithm delivers a significantly lower power of 392 W from the PV system. Both FPA and CSA achieve a power of 528.74 W with a tracking time of 0.23 s, showing similar performance. The IGWO algorithm reaches 528.26 W in only 0.11 s, achieving nearly the same efficiency but with a much higher tracking speed.

**Fig. 10:**
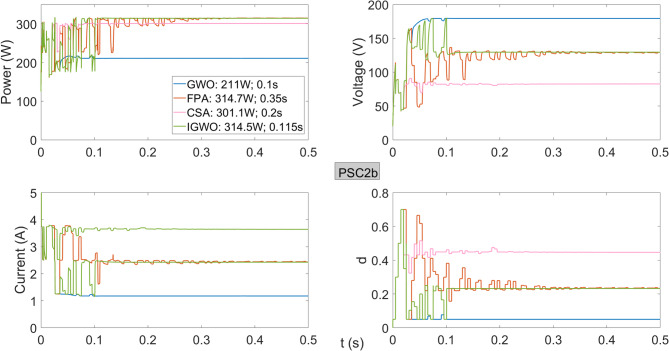
Power, current, voltage, and duty cycles obtained using GWO, FPA, CSA, and IGWO algorithms for PV system under PSC2b conditions.

As shown in Fig. 10, under PSC2b, the highest powers are obtained using FPA and IGWO, with values of 314.7 W and 314.5 W, respectively. In terms of tracking speed, IGWO reaches the maximum power in 0.115 s, whereas FPA requires 0.35 s, clearly demonstrating the speed advantage of IGWO. For the voltage regions corresponding to the maximum power obtained under PSC2, PSC2a, and PSC2b conditions, FPA produces slightly higher power than IGWO; however, both algorithms achieve approximately the same maximum power. On the other hand, IGWO stands out due to its superior tracking speed.

**Fig. 11:**
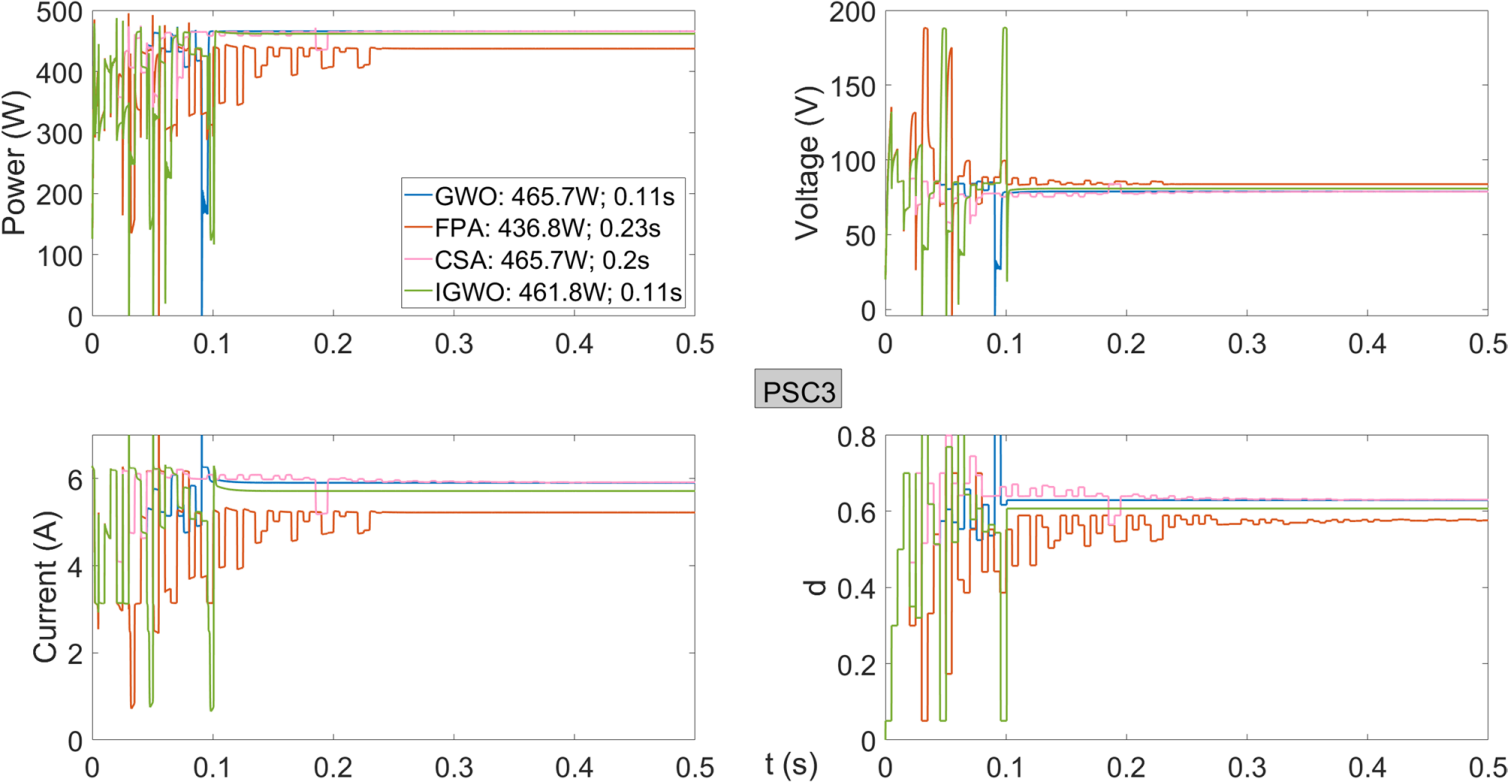
Power, current, voltage, and duty cycles obtained using GWO, FPA, CSA, and IGWO algorithms for PV system under PSC3 conditions.

Figure 11 shows the power, voltage, current, and duty cycles obtained from the PV system operating under PSC3. Compared to other voltage regions, the FPA algorithm delivers a lower power of 436.8 W, indicating reduced performance. The IGWO algorithm achieves 461.8 W, operating at slightly lower efficiency than FPA and CSA in this scenario. In terms of tracking speed, both GWO and IGWO reach the maximum power in 0.11 s, performing significantly better than the other two algorithms.

**Fig. 12:**
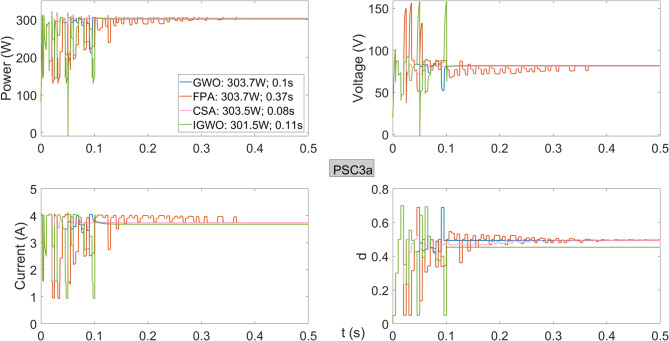
Power, current, voltage, and duty cycles obtained using GWO, FPA, CSA, and IGWO algorithms for PV system under PSC3a conditions.

Figure 12 presents the power, voltage, current, and duty cycles obtained from the PV system operating under PSC3a conditions using the different algorithms. While GWO, FPA, and CSA achieve approximately the same power from the PV system, IGWO delivers a lower power of 301.5 W. In this voltage region, the GWO algorithm demonstrates superior performance in terms of both efficiency and tracking speed.

**Fig. 13:**
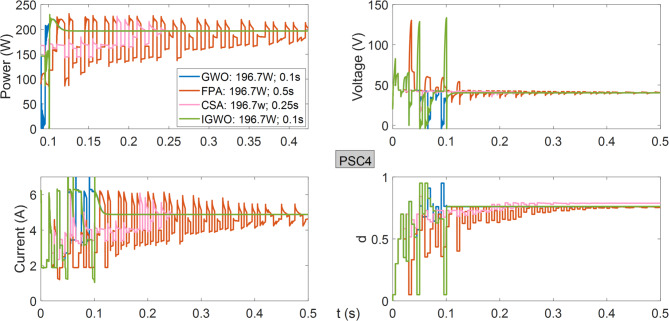
Power, current, voltage, and duty cycles obtained using GWO, FPA, CSA, and IGWO algorithms for PV system under PSC4 conditions.

As observed in Fig. 13, all algorithms achieve the same power from the PV system operating under PSC4. Both GWO and IGWO algorithms demonstrate successful performance with a tracking speed of 0.1 s.

**Fig. 14:**
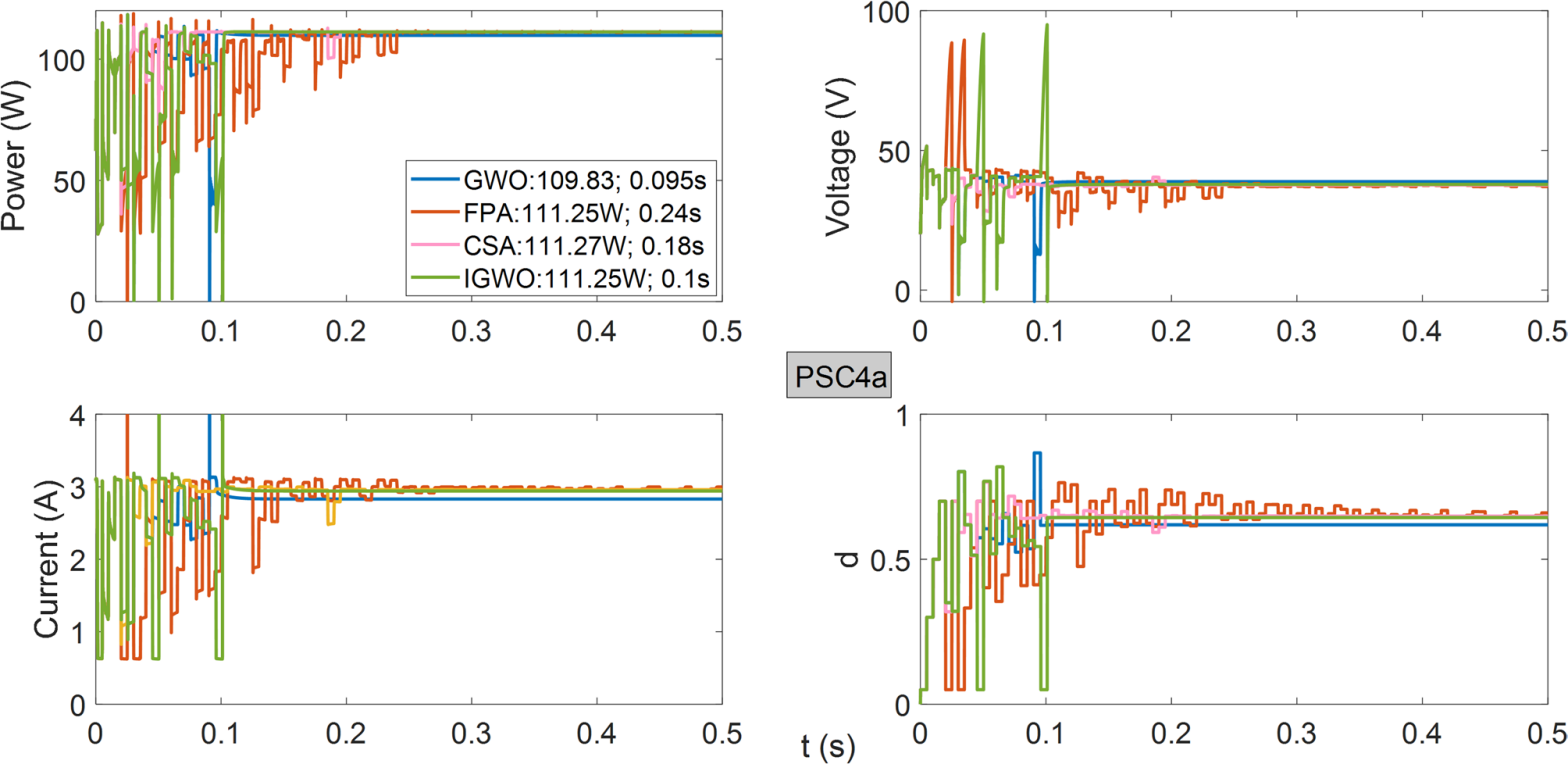
Power, current, voltage, and duty cycles obtained using GWO, FPA, CSA, and IGWO algorithms for PV system under PSC4a conditions.

As shown in Fig. 14, the lowest maximum power generated in the lowest voltage region is obtained using the GWO algorithm. FPA, CSA, and IGWO produce approximately the same power, while the IGWO algorithm stands out due to its superior tracking speed.

The powers, efficiencies, and tracking speeds obtained under all atmospheric conditions are presented in Table 4. The efficiency of the PV system is calculated as seen in Eq. (11).

11 $$\eta \% = \frac{{P_{pv} }}{{P_{GMPP} }} \times 100$$

where, $P_{pv}$ represents the power obtained from the PV system and is calculated as $V_{pv} \times I_{pv}$. $P_{GMPP}$ represents the maximum power that can be obtained in the PSC scenario and is determined using P–V curves as shown in Fig. 5. Tracking speeds of the MPPT algorithms is calculated as in shown Eq. (12). Each iteration occurred at 5 ms intervals. The sampling time of the duty period is 25 µs and is compatible with the switching frequency of the boost converter.

12 $$t_{s} = \frac{{\left| {P_{pv} - P_{GMPP} } \right|}}{{P_{GMPP} }} < 0.5$$

**Table 4:** Powers, efficiencies, and tracking speeds obtained from nine different PSC scenarios using GWO, FPA, CSA, and IGWO algorithms.

	Maximum power (W)				Efficiency (%)				Tracking speed (s)			
	GWO	FPA	CSA	IGWO	GWO	FPA	CSA	IGWO	GWO	FPA	CSA	IGWO
PSC1	584.1	625.4	524.88	623.01	93.44	100	84.6	99.66	0.1	0.24	0.2	0.11
PSC1a	220.1	305	235.2	306	71.96	99.72	76.9	100	0.1	0.48	0.21	0.12
PSC2	461.8	465.1	465.1	464	99.3	100	100	99.78	0.1	0.37	0.18	0.11
PSC2a	392	528.74	528.74	528.26	73.49	99.13	99.13	99.04	0.1	0.23	0.23	0.11
PSC2b	211	314.7	301.1	314.5	67.12	100	95.78	100	0.1	0.35	0.2	0.115
PSC3	465.7	436.8	465.7	461.8	99.94	93.74	99.94	99.11	0.11	0.23	0.2	0.11
PSC3a	303.7	303.7	303.5	301.5	99.53	99.53	99.47	98.81	0.1	0.37	0.08	0.11
PSC4	196.7	196.7	196.7	196.7	88.96	88.96	88.96	88.96	0.1	0.5	0.25	0.1
PSC4a	109.83	111.25	111.27	111.25	98.58	99.85	99.85	99.85	0.09	0.24	0.18	0.1
Mean	327.21	**365.26**	348.02	**367.45**	88.03	**97.88**	93.85	**98.34**	**0.1**	0.33	0.19	**0.11**

Examining Table 4, the average power obtained across all scenarios is highest with the IGWO algorithm, reaching 367.45 W. The FPA algorithm achieves 365.26 W, coming very close to the IGWO result. This trend is also reflected in the average efficiencies, with FPA and IGWO achieving 97.88% and 98.34%, respectively, which are very similar. A notable difference is observed in tracking speeds: the average tracking time of IGWO is 0.11 s, whereas FPA requires 0.33 s. Thus, in addition to the small advantage in efficiency, IGWO reaches the maximum power approximately three times faster than FPA. The challenging selection of atmospheric conditions and the wide duty cycle range over which power is obtained further demonstrate the robustness and effectiveness of the proposed algorithm.

**Fig. 15:**
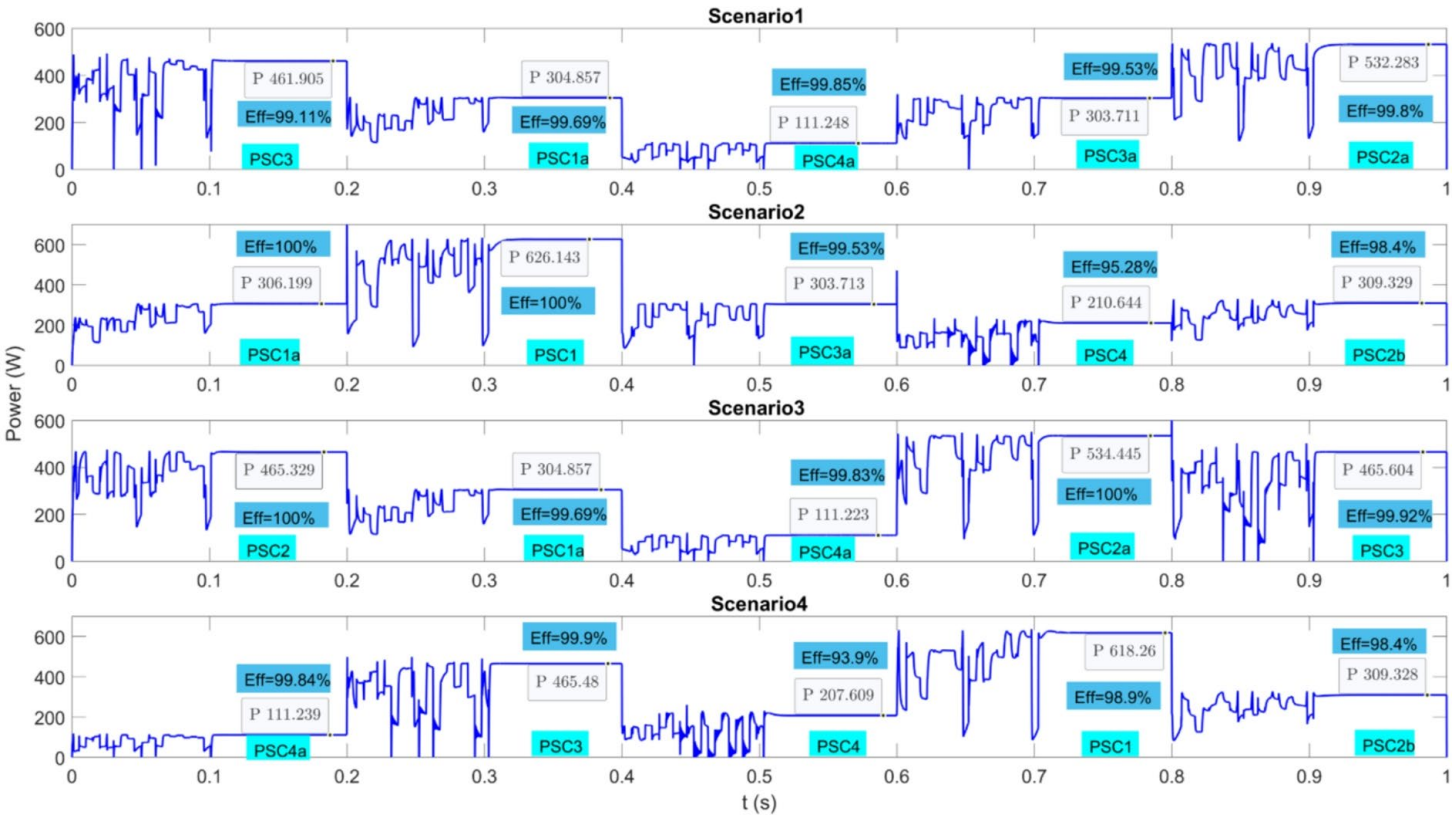
Power obtained from a PV system operating under four different dynamic atmospheric conditions using the IGWO method.

To evaluate the performance of the proposed algorithm under rapidly varying atmospheric conditions, four different scenarios were designed, as illustrated in Fig. 15. In each scenario, five distinct PSCs states were applied to the PV system at 0.2 s intervals using different combinations. The steady-state power values and the corresponding efficiencies obtained for each PSC state are presented in the figure. Accordingly, it was observed that high-efficiency operation was achieved in nearly all cases. In conclusion, the superior performance of the proposed MPPT algorithm under dynamic atmospheric conditions has been demonstrated.

In Scenario 1, considering the steady-state algorithm efficiencies of five different PSC conditions, a very good average efficiency of 99.6% was obtained. In Scenario 2, the average efficiency of 5 different PSCs applied sequentially was obtained as 98.64%. However, only PSC4 showed low efficiency. Currently, the efficiency of all algorithms under PSC4 is very low. In other cases, the efficiency is quite high. In Scenario 3, the average efficiency was very high at 99.89%. In Scenario 4, the average efficiency was very high at 98.12%. However, the efficiency is again reduced by the operation under PSC4. Very high efficiency was achieved under other atmospheric conditions.

Figure 15 shows that the proposed algorithm is as successful as other methods when the simulations are repeated. When the system is first energized, the voltage rise times can change in transient situations due to the system’s behavior. The duty cycles recorded in the first study can sometimes give misleading results. As seen in Fig. 15, higher power was obtained in PSC3a in Scenario 1 and Scenario 2. Overall, the superiority of the method is clearly seen in the average power obtained from nine different scenarios.

The high speed and efficiency of the IGWO method have been demonstrated through simulation studies. On the other hand, a significant advantage of the IGWO algorithm over other modified GWO and hybrid GWO algorithms is its simple structure. In the proposed approach, if steady-state operation cannot be achieved within the first 10 iterations, the initial conditions are adjusted using a simple procedure and the GWO is restarted. In^36^, the convergence speed was improved by deriving the convergence factor through an alternative formulation; however, the use of complex mathematical operations increased the overall complexity of the optimization method. In^37^, a nonlinear convergence factor calculation method was proposed, and the newly introduced approach involving trigonometric expressions further increased the computational burden. In^38–43^, new hybrid optimization algorithms were developed by combining two different optimization techniques. The computational complexity of such hybrid algorithms is inevitably high. A review of the literature indicates that the IGWO method stands out due to its high efficiency, rapid convergence, and low computational complexity.

## Conclusion

Tracking the global maximum power point rapidly and with high efficiency remains a major challenge for MPPT algorithms in PV systems operating under partial shading conditions (PSC). Therefore, the development of new MPPT algorithms that simultaneously improve tracking speed and ensure high steady-state efficiency is of great importance. In this study, a PV system consisting of four series-connected PV panels and a boost converter was modeled in the MATLAB/Simulink environment, and nine different PSC scenarios were constructed to evaluate the performance of MPPT algorithms. The widely used GWO algorithm was improved by redefining its operating boundaries, resulting in a novel Improved Grey Wolf Optimization (IGWO)-based MPPT algorithm that offers both high tracking speed and high efficiency. The performance of the proposed algorithm was comparatively analyzed against well-established algorithms in the literature, namely the Flower Pollination Algorithm (FPA) and the Cuckoo Search Algorithm (CSA). The IGWO algorithm was obtained through a simple yet effective modification of the conventional GWO approach. By restricting the search space and restarting the algorithm after a predefined number of iterations, the hunting (search) capability was enhanced, enabling faster convergence toward the maximum power point. To ensure a fair comparison, both the conventional GWO and the proposed IGWO algorithms were executed using the same number of iterations. The results demonstrate that the IGWO algorithm provides a significantly higher average tracking efficiency compared to the conventional GWO. Across the nine PSC scenarios, the IGWO algorithm achieved an average power of 367.45 W, while the FPA algorithm yielded a closely comparable average power of 365.26 W. In terms of average tracking efficiency, IGWO and FPA achieved 98.34% and 97.88%, respectively. However, a notable distinction was observed in tracking speed: the IGWO algorithm reached the maximum power point with an average tracking time of 0.11 s, whereas the FPA algorithm required 0.33 s. These findings indicate that, in addition to delivering high efficiency, the IGWO algorithm offers approximately three times faster tracking performance.

In conclusion, this study introduces a new IGWO-based MPPT algorithm capable of rapidly and efficiently extracting maximum power from PV systems operating under partial shading conditions. Simulation results obtained under challenging PSC scenarios clearly confirm the effectiveness and reliability of the proposed method. Future work will focus on supporting the IGWO algorithm with hybrid structures and further improving its average tracking efficiency, aiming to develop next-generation MPPT algorithms that simultaneously provide very high speed and ultra-high efficiency.

## Data Availability

The datasets used and/or analysed during the current study available from the corresponding author on reasonable request.
